# Progressive Pulmonary Artery Dilatation is Associated with Type B Aortic Dissection in Patients with Marfan Syndrome

**DOI:** 10.3390/jcm8111848

**Published:** 2019-11-02

**Authors:** Christel Brouwer, Haldun Bulut, Willemijn van Gemert, Alexander HJ Staal, Kim Cortenbach, Miranda Snoeren, Robin Nijveldt, Anthonie Duijnhouwer, Bart L Loeys, Niels van Royen, Janneke Timmermans, Roland RJ van Kimmenade

**Affiliations:** 1 Department of Radiology and Nucleair Imaging, Radboud University Medical Center, Nijmegen 6500 HB, the Netherlands; brouwer.christel@gmail.com (C.B.); willemijn.vangemert@radboudumc.nl (W.v.G.); miranda.snoeren@radboudumc.nl (M.S.); 2 Department of Cardiology, Radboud University Medical Center, Nijmegen 6500 HB, the Netherlands; haldun.bulut@radboudumc.nl (H.B.); kim.cortenbach@radboudumc.nl (K.C.); robin.nijveldt@radboudumc.nl (R.N.); toon.duijnhouwer@radboudumc.nl (A.D.); niels.vanroyen@radboudumc.nl (N.v.R.); janneke.timmermans@radboudumc.nl (J.T.); 3 Department of Clinical Genetics, Radboud University Medical Center, Nijmegen 6500 HB, the Netherlands; bart.loeys@radboudumc.nl; 4 Center for Medical Genetics, Antwerp University Hospital & University of Antwerp, Antwerp 2000, Belgium

**Keywords:** aorta, Marfan, dissection, diagnosis

## Abstract

Objective: Marfan syndrome (MFS) is a connective tissue disorder associated with severe cardiovascular morbidity and mortality. It is unknown if aorta complications in MFS are associated with progressive pulmonary artery (PA) dilatation. Methods: We measured the PA diameter on routine magnetic resonance imaging in a population of MFS patients seen in our specialised centre with follow up of diameters as well as the outcome. Results: PA dilatation was defined as an increase in diameter of 2 mm or more, and 71 patients (44%) of our total cohort (*n* = 162) met this criterion; mean follow up between two scans was 8.6 years (standard deviation (SD) ± 2.7 years). Furthermore, 28 patients suffered from dissections, of which 14 had a type A dissection, 10 had a type B dissection, and 4 patients suffered from both. Of those who suffered from dissection, 64% (18 out of 28) had a dilatation of the PA, versus 39% (53 out of 134) in the patient group without a dissection (*p* < 0.05). There was a significant association between type B dissection and descending aorta diameter (OR 1.14; 95% CI 1.05–1.24 *p* < 0.01) and PA dilatation (OR 1.69; 95% CI 1.03–2.77 *p* = 0.04). In the multivariable analysis the final model for type B dissection, only systolic blood pressure (OR 1.06; 95% CI 1.01–1.11 *p* = 0.02) and PA dilatation were statistically significant (OR 1.85; 95% CI 1.10–3.12 *p* = 0.02) while descending aorta diameter was not. Conclusions: We report an association between progressive PA dilatation and type B dissection. Our findings encourage a renewed interest in PA dimensions in MFS.

## 1. Introduction

Marfan syndrome (MFS) is an autosomal dominant connective tissue disorder, in which cardiovascular complications and more specific aortic dissections are associated with severe morbidity and mortality [1]. Type A aortic dissection especially, i.e., aortic dissection propagating from the ascending aorta, is the most life threatening due to cardiac tamponade, acute severe aortic regurgitation, and/or coronary or cerebral artery involvement. Although type B dissection, i.e., dissection starting distal from the subclavian artery, does not necessarily result in life-threatening complications, in some cases, immediate surgery or intervention is, however, warranted in order to preserve vital organ function [2,3]. Importantly, long term prognosis in type B is actually worse than in type A [4].

Medical interest in MFS is, thus, focused on the aorta, and although involvement of the pulmonary artery (PA) has been studied previously, it has lost clinical relevance. To illustrate this, in the revised Ghent nosology introduced in 2010, more diagnostic weight was given to fibrillin-1 mutation (FBN-1), as well as aortic root dimensions and ectopia lentis, while dilatation of the main pulmonary artery (PA) was excluded from the diagnostic criteria [5]. The reason for the latter was that PA dilatation was considered to be non-specific to MFS and complications in the PA occur rarely.

Actual documentation and follow-up data of PA dilatation are, however, sparse. Yet, since the PA and aorta originate from the same common trunk but are exposed to other pressures and haemodynamics after birth, we hypothesised that the follow up of PA dimensions might be of clinical relevance since it may reflect tissue characteristics independent of blood pressure exposure and altered flow patterns [6]. We, therefore, studied the longitudinal follow-up of PA dimensions and its association with vascular complications in a large MFS population.

## 2. Methods

### 2.1. Patients

Our centre is a tertiary referral centre for MFS. All patients are seen on an annual base as published previously, and consecutively prospectively entered in our database [7]. All patients used for the present study had to have a proven FBN1 mutation diagnostic for MFS, and have undergone two or more MRI (magnetic resonance imaging) scans. Our registry has been approved by the local Medical Ethical Committee.

### 2.2. Magnetic Resonance Imaging

All MRI images were acquired on a 1.5T clinical MRI scanner (Siemens Vision/Avanto, Siemens Healthcare GmbH, Erlangen, Germany) using aortic diameter protocol without intravenous contrast or MRA protocol with intravenous contrast (gadolinium), all with a slice thickness of 8 mm. Aortic and PA diameters were measured on the axial and sagittal multislice balanced steady-state free precession images (typical voxel size 2.0 × 1.3 × 8 mm^3^) or gadolinium-enhanced MRA (typical voxel size 1.5 × 1 × 1 mm^3^).

### 2.3. Measurements

**Figure 1:**
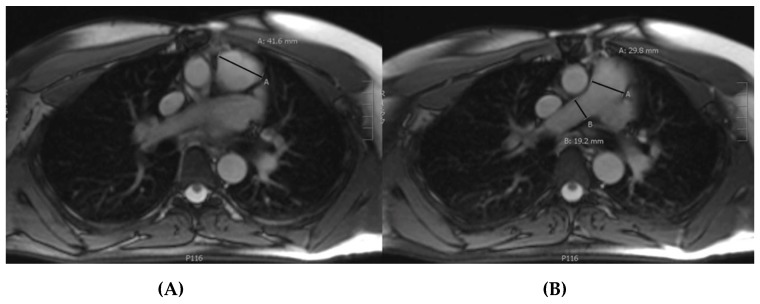
Measurement of the pulmonary root (**A**), pulmonary trunk and right pulmonary artery (**B**) on axial images in a balanced steady-state free precession sequence without contrast.

All measurements were performed by the same observer and blinded for the outcome. In every patient, eight inner to inner diameters were measured at two time-points. These included the following diameters on the axial images: the aortic root, ascending aorta at the level of the pulmonary bifurcation, descending aorta at the level of the left atrium, pulmonary root, pulmonary trunk, right pulmonary artery, and left pulmonary artery. On the sagittal images, the aortic arch just distal of the left subclavian artery was measured. In measuring the aortic root, ascending aorta, and descending aorta, the largest diameter was measured. Figure 1 shows three of the four measurements in the PA. In some cases, there was a dilatation of the descending aorta at another location than at the level of the left atrium, which was measured separately.

### 2.4. Statistics

Baseline descriptive patient characteristics were provided for the study group as a whole and per type of dissection. Data are presented as medians and interquartile ranges (IQR) for continuous variables or numbers and percentages for categorical variables. Possible explanatory factors were selected based on reasoning/hypothesis. For these factors, univariate correlations with PA growth were examined using logistic regression analyses in patients with a type A or B dissection or both as a reference category. All variables from the univariate associations were entered in a multivariable full model. Backward model selection was then performed wherein a *p*-value of 0.10 was used for variable selection. We compared the odds ratios and standard deviations after every step, there were no signs of multicollinearity, and data were analysed using SPSS Statistics (version 22.0, IBM SPSS Inc, Armonk, New York, NY, USA).

## 3. Results

At the moment of analysis, 181 patients were diagnosed with an FBN-1 mutation, of which 162 had undergone two or more MRI scans. The mean follow up time between two scans was 8.6 years (standard deviation (SD) ± 2.7 years).

In this cohort (*n* = 162), 28 patients suffered from aortic dissections, of which 14 had a type A dissection (mean age 42.8 years; SD ± 9.0 years), 10 had a type B dissection (mean age 36.6 years; SD ± 12.6 years), and four patients suffered from both (mean age 33.9 years; SD ± 10.2 years). A total of 76 (46.9%) patients underwent aortic surgery, of which 48 underwent elective surgery. No patients underwent PA surgery.

In the overall group, 82.1% (*n* = 133) of the patients were treated medically with a beta-blocker (88.9% and 92.9% of the patients in the type A and B dissection groups, respectively), of which 2.3% (*n* = 3) were combined with an aldosterone receptor antagonist and 2.3% (*n* = 3) with a calcium antagonist. A total of 47.5% (*n* = 77) of the patients were treated with an angiotensin-1 receptor blocker (55.6% and 57.1% of the patients in the type A and B dissection groups, respectively. Other baseline characteristics of our study population are given in Table 1.

**Figure 2:**
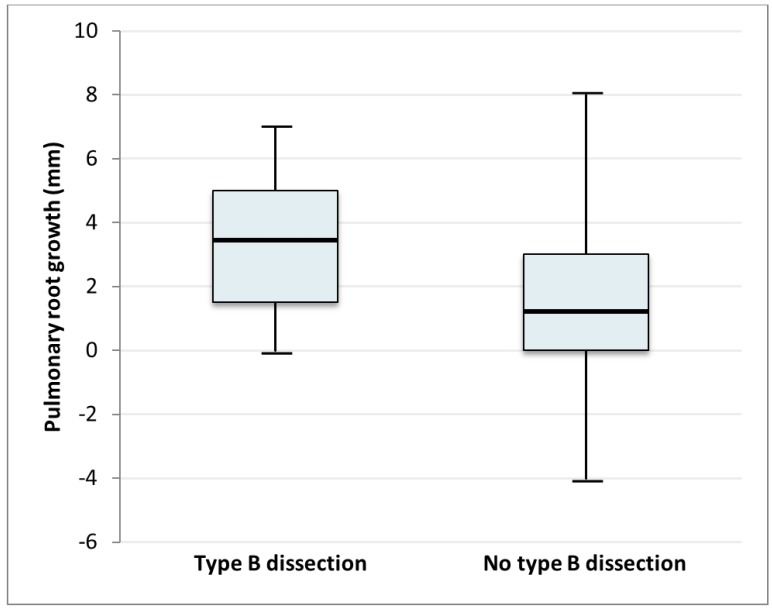
Pulmonary root growth in mm in patients with type B dissection (median 3.5, interquartile range (IQR) 1.5–5) and in patients without type B dissection (median 1, IQR 0–3). *p* < 0.05.

PA dilatation was defined as a difference in diameter of 2 mm or more. This cut-off was chosen arbitrarily due to the lack of an established definition and since 2 mm is above any variability in measurement. A total of 71 patients (43.8%) met this criterion. Of the patients who had a dissection, 64% (18 out of 28) had a growth of the PA, versus 39% (53 out of 134) in the patient group without a dissection. The median PA growth was 3.5 mm, and the interquartile range (IQR) was 1.5–5 mm in those who suffered from type B dissection, while the median growth was 1 mm (IQR 0–3mm) in those who did not (Figure 2).

There was a significant association between type A dissection and the variables age (odds ratio, OR, 1.04), diameter of the ascending aorta (OR 1.15), and aorta dilatation rate (OR 0.31). PA dilatation was not significantly associated (*p*-value = 0.50). For a type B dissection, the variables descending aorta diameter (OR 1.14) and pulmonary root dilatation (OR 1.69, *p* = 0.04) showed a significant association. In the group of patients with a dissection (both or either type A or B), the variables sex (OR 2.11), age (OR 1.03), systolic blood pressure (OR 1.03), aorta ascendens diameter (OR 1.13) and aorta descendent diameter (OR 1.27) showed significant associations. Again, pulmonary root dilatation was not significantly associated (*p* = 0.17). Data are shown in Table 2.

In the multivariable analysis, the final model for type A dissection did not contain pulmonary root dilatation. The model for type B dissection contained systolic blood pressure (OR 1.06) and pulmonary root dilatation (OR 1.85), which were both statistically significant (both *p*-value of 0.02). The final model for the group with a dissection (all types) contained systolic blood pressure (OR 1.04, *p* = 0.11), aorta descendens diameter (OR 1.17, *p* = 0.04) and pulmonary root dilatation (OR 1.50, *p* = 0.09). The R^2^, or explained variance, was highest for the model concerning all dissection types (R^2^ = 0.345). Figure 2 shows the differences in pulmonary artery dilation in patients with or without a type B dissection.

## 4. Discussion

Marfan syndrome (MFS) is an autosomal dominant connective tissue disorder, in which cardiovascular complications and especially type A dissection are the most life threatening, thus gaining the most clinical interest.

The first official definition of MFS was described in the Berlin criteria in 1986 and was based only on clinical phenotype such as skeletal, aortic, and pulmonary features [8]. In 1996, the Ghent I nosology was developed, in which the presence of mutations in the FBN1 gene was implemented as a major diagnostic criterion, while dilatation of the pulmonary root under the age of 40 became a minor criterion for establishing the diagnosis of MFS [9]. With the revised Ghent nosology introduced in 2010, even more diagnostic weight was given to aortic root aneurysm and/or dissection (and ectopia lentis), while dilatation of the main pulmonary artery was completely excluded since this was considered non-specific and without clinical relevance since PA complications in MFS are rare [5]. Hence, the cardiovascular focus in MFS is now completely on aorta and aortic dimensions.

The prognosis in patients suffering from MFS has improved significantly since the introduction of the Bentall procedure (i.e., composite graft implantation in aortic position) in 1968 because type A dissection can now be treated or prevented [10,11]. The incidence of type A dissection seems mainly related to the diameter of the aortic root, and the widest aortic diameters are considered to have the highest risk for type A dissection, according to the law of Laplace [12]. Although type B dissection does not necessarily result in life-threatening complications, in some cases, urgent endovascular procedures or surgery are warranted in order to prevent, for example, systemic organ failure due to hypoperfusion or retrograde progression of the dissection. Interestingly, although guidelines advocate for intervention in patients (without connective tissue disorders) with a descending aorta above 50 mm [13], there is only a poor association between descending aorta dimensions and the occurrence of type B dissections. In fact, 80% of the patients suffering from a type B dissection had a descending aorta diameter below the threshold for intervention [14], and the international consensus is that descending aorta diameter is an inferior predictor for type B dissection. Interestingly, Shirali et al. found that ascending aorta dimensions better predict type B dissection in hypertensive subjects, while den Hartog et al. found that a proximal descending aorta diameter ≥27 mm is the best predictor of type B dissection. Here, we show that the association between dilatation of the PA and type B dissection is stronger than between ascending or descending aorta dimensions and type B dissections [15,16].

Up until now, no longitudinal data on PA dilatation in adult patients with MFS are available. Nollen et al. showed significant higher PA dimensions in Marfan patients than in controls, and within the Marfan group, a significant larger pulmonary root in patients with aortic root replacement. Also, they found that the difference in diameter between the pulmonary root and the pulmonary bifurcation is larger in Marfan patients. They used the anterior-right diameter of the pulmonary root on MRI because this dimension showed the smallest variability [17]. In contrast, Sheikhzadeh et al. found no difference in PA diameter between persons with and without MFS using echocardiography, although they did find an increase in the ratio between PA diameter and aortic root. In addition, approximately 70% of MFS patients suffered from the mean PA dilatation and 15% from the mean PA aneurysm [18]. Lundby et al. found that previous surgery on the ascending aorta is associated with dilatation of the trunk of the PA but not with dilatation of the root [19]. More recently, Stark and co-workers also found that dilated PA diameters on echocardiography in children are associated with an earlier occurrence of aortic dilatation, mitral valve prolapse, and systemic manifestations of MFS. In conclusion, there seems to be an association between pulmonary artery dimensions and aortic root involvement in Marfan patients, but longitudinal data are lacking [20].

The PA and aorta derive from the same embryological origin, the so-called ‘common trunk’ which is split in order to form the ascending aorta and the PA [6]. This implicates that after birth, intrinsic tissue characteristics of both vessels may remain the same but that these ‘twin’ vessels are now exposed to other blood pressures and haemodynamics. We, therefore, investigated the follow up of PA dimensions and its association with aortic complications in a large MFS population.

Several limitations should be taken into account when interpreting this study. Firstly, our data are only observational, and it cannot be concluded from our data that descending aorta interventions in patients with progressive PA dilatation already should be performed earlier. Secondly, only patients with MFS with the FBN-1 mutation were used in this analysis. Therefore, data cannot be extrapolated to other connective tissue disorders or hypertension patients. Thirdly, PA measurements were not performed doubly oblique, but since all measurements were performed in the same manner as described above, the reported association remains valid. However, PA dilatation is an easily available diagnostic tool since all subjects with MFS undergo routinely aortic imaging, and by including PA dilatation in this follow up, physicians may better risk-stratify MFS patients.

In conclusion, we now, for the first time, show a longitudinal follow up of PA dimensions in MFS. Progression of PA dilatation is associated with the occurrence of type B dissection, which might reflect tissue vulnerability. Further investigation is needed in order to stratify the clinical relevance of progressive PA dilatation, while we advocate measuring PA dimensions in routine follow up of aorta dimensions in MFS.

### Key messages

What is already known about this subject?

—Marfan syndrome has high cardiovascular morbidity, especially due to aortic dissections.

What does this study add?

—We describe that progressive pulmonary artery dilatation is associated with type B dissection, with a better odd’s ratio than all present predictors.

How might this impact on clinical practice?

—Measurement of the pulmonary artery, available at no extra costs in the routine work-up may help in better risk estimation in Marfan syndrome.

## 5. Conclusions

We show for the first time a longitudinal follow up of PA dimensions in MFS. Progression of PA dilatation is associated with the occurrence of type B dissection, which might reflect tissue vulnerability.

Further investigation is needed in order to define the clinical relevance of progressive PA dilatation, while we support measuring of PA dimensions in routine follow up of aorta dimensions in MFS.

## Figures and Tables

**Table 1:** Baseline characteristics. Pulmonary root dilatation: number of patients with a dilatation of the pulmonary root, equal to or more than 2 mm, between the first and last MRI. There was missing data on BMI, aorta ascendens, aorta descendens, pulmonary bifurcation (*n* = 1 subject); body weight, systolic blood pressure, diastolic blood pressure (*n* = 2); aortic root, aortic arch, right pulmonary artery (*n* = 3); pulmonary root (*n* = 6), and left pulmonary artery (*n* = 8).

	Whole Population (*n* = 162)	Type B Dissection (*n* = 10)	Type A Dissection (*n* = 14)	Both Type A and B (*n* = 4)	No Dissection (*n* = 134)
**Baseline Characteristics**					
Male, number (%)	86 (53.1%)	7 (70%)	11 (79%)	1 (25%)	67 (50.0%)
Age (y), mean (SD)	34.7 (12.8)	36.6 (12.6)	42.8 (9.0)	33.9 (10.2)	33.8 (13.0)
Body weight (kg), mean (SD)	81.5 (18.2)	84.1 (14.2)	91.8 (19.0)	83.3 (25.4)	80.1 (18.0)
BMI, mean (SD)	23.3 (5.3)	22.6 (2.6)	26.3 (5.8)	23.2 (7.0)	23.0 (5.3)
Systolic blood pressure (mmHg), mean (SD)	125.8 (17.2)	134.7 (23.7)	132.5(12.1)	129.5 (20.8)	124.3 (16.8)
Diastolic blood pressure (mmHg), mean (SD)	75.4 (10.5)	75.5 (11.6)	76.9 (9.4)	74.3 (4.5)	75.3 (10.7)
Time of follow-up (between two MRI’s, years)	8.6 (2.7)	10.9 (1.6)	8.9 (2.9)	7.5 (1.9)	8.4 (2.7)
**Aorta and Pulmonary Artery Diameters**					
Aortic root (mm), mean (SD)	34.7 (5.4)	36.7 (4.8)	31.4 (6.2)	32.5 (2.9)	35.0 (5.3)
Aorta ascendens (mm), mean (SD)	28.5 (4.7)	29.5 (5.6)	33.2 (5.8)	26.3 (2.2)	28.0 (4.3)
Aortic arch (mm), mean (SD)	22.3 (3.5)	23.1 (1.6)	27.7 (5.0)	22.3 (1.5)	21.6 (2.9)
Aorta descendens (mm), mean (SD)	21.4 (4.8)	27.3 (8.6)	26.6 (6.0)	21.3 (2.1)	20.4 (3.6)
Pulmonary root (mm), mean (SD)	32.8 (4.5)	35.3 (4.1)	35.9 (2.7)	31.5 (4.7)	32.4 (4.5)
Pulmonary bifurcation (mm), mean (SD)	25.0 (3.4)	24.8 (1.9)	27.7 (1.5)	25.8 (6.4)	24.7(3.4)
Right pulmonary artery (mm), mean (SD)	16.6 (3.2)	17.6 (2.8)	18.6 (2.9)	14.5 (2.5)	16.3 (3.2)
Left pulmonary artery (mm), mean (SD)	17.2 (2.9)	17.7 (2.4)	19.8 (3.0)	18.8 (3.9)	16.8 (2.8)
Pulmonary root dilatation ≥2 mm	71 (43.8%)	7 (70.0%)	7 (50.0%)	4 (100%)	53 (39.6%)

**Table 2:** Logistic regression models. Univariate associations and multivariate associations of the best fitted models are presented. All variables were entered in the full model. Backward model selection was then performed wherein a *p*-value of 0.10 was used for variable selection. Pulmonary root dilatation is defined as growth ≥2 mm (continuous variable). Odds ratios (OR) with 95% confidence interval (95% CI). For interpretation: the outcome odds ratio approaches a relative risk, based on rare incidence of dissection. ^R^2^: proportion of variance in the dependent variable that is explained by the independent variable(s).

	Univariate				Multivariable			
	OR	95% CI	*p*-Value	^R^2^	OR	95% CI	*p*-Value	^R^2^
**Type A Dissection**								0.074
Sex (male)	1.89	0.67; 5.32	0.23	0.019				
Age (years)	1.04	1.003; 1.08	0.04	0.054				
Systolic blood pressure (mmHg)	1.02	1.00; 1.05	0.12	0.029				
Diameter ascending aorta (mm)	1.15	1.04; 1.27	<0.01	0.098	1.11	1.00; 1.24	0.08	
Pulmonary root dilatation (mm)	1.18	0.73; 1.92	0.50	0.011				
**Type B Dissection**								0.265
Sex (male)	1.20	0.40; 3.62	0.75	0.001				
Age (years)	1.01	0.97; 1.05	0.74	0.002				
Systolic blood pressure (mmHg)	1.03	0.10; 1.01	0.09	0.037	1.06	1.01; 1.11	0.02	
Diameter descending aorta (mm)	1.14	1.05; 1.24	0.003	0.114				
Pulmonary root dilatation (mm)	1.69	1.03; 2.77	0.04	0.108	1.85	1.10; 3.12	0.02	
**Both or Either**								0.345
Sex (male)	2.11	0.89; 5.00	0.09	0.031				
Age (years)	1.03	1.00; 1.01	0.04	0.043				
Systolic blood pressure (mmHg)	1.03	1.00; 1.051	0.02	0.056	1.04	1.00; 1.10	0.11	
Diameter ascending aorta (mm)	1.13	1.04; 1.23	0.005	0.081				
Diameter descending aorta (mm)	1.27	1.14; 1.41	<0.001	0.267	1.17	1.01; 1.37	0.04	
Pulmonary root dilatation (mm)	1.34	0.88; 2.04	0.17	0.039	1.50	1.00; 2.40	0.09	
